# The onset of data-driven mental archeology

**DOI:** 10.3389/fnins.2014.00249

**Published:** 2014-08-13

**Authors:** Sidarta Ribeiro

**Affiliations:** Laboratory of Memory, Sleep and Dreams, Brain Institute, Federal University of Rio Grande do NorteNatal, Brazil

**Keywords:** mind, self concept, semantic holism, Julian Jaynes, semantic distance, bronze age

How did human consciousness arise, in comparison with that of apes? This question has motivated many hypotheses, from psychoanalysis (Freud, 1939) and literature (Bloom, 1999) to evolutionary psychology (Dennett, 1997; Tomasello, 2014). One of the most provocative is that of Julian Jaynes (1976), who understood the question as pertaining to the cultural evolution of introspection during the first millennium BC, known as Axial Age (Jaspers, 1953). Based on ancient texts, Jaynes postulated that our current awareness began with memories of commands uttered by clan chiefs, which allowed for sustained work throughout the day even in the chief's absence. When chiefs died the memories of their voices reverberated in the remaining subjects, and this occurred more strongly in dreams than in waking, due to the absence of sensory interference. Humans evolved a bicameral mind in which part of the activity dealt with the present to perform actions, while the other part dealt with past and future to produce auditory hallucinations perceived as external commands.

Over millennia, people with such mental organization left the caves to build cities and pyramids, as societies with hundreds of thousands members led by a few who heard the voices of dead ancestors turned gods. Eventually there were too many mouths to feed, the empires collapsed, and the voices became mute. Texts spanning several centuries before 1000 BC reflect this divine silence, as the mental separation between gods and people gradually dissolved. The bicameral person who heard voices gave place to the unicameral individual in whom the self-representation uses its vast mnemonic repertoire not to hallucinate, but to imagine plans. For contemporary mentality the incessant voices of our internal dialog are ours only, not from other entities. Those who today are still split in mental compartments would be psychotic.

For many years this shrewd hypothesis seemed untestable. Corollaries such as the right lateralization of auditory hallucinations were dismissed as too simplistic—although schizophrenic patients present less language lateralization (Sommer et al., 2001). Yet, the investigation by Diuk et al. (2012) represents a pioneering successful attempt to test Jaynes' theory in a quantitative manner. The authors assessed dozens of Judeo-Christian and Greco-Roman texts from up to the second century CE, as well contemporary Google n-grams, to calculate semantic distances between the reference word “introspection” and all the words in these texts. Cleverly, “introspection” is actually absent from these ancient texts, serving as an “invisible” probe. Semantic distances were evaluated by Latent Semantic Analysis, a high-dimensional model in which the semantic similitude between words is proportional to their co-occurrence in texts with coherent topics (Deerwester et al., 1990; Landauer and Dumais, 1997). The approach goes well beyond the mere counting of word occurrence in a corpus, actually measuring how much the concept of introspection is represented in each text in a “distributed semantic sense,” in accordance with the semantic holism (Frege, 1884, 1980; Quine, 1951; Wittgenstein, 1953, 1967; Davidson, 1967) that became mainstream in artificial intelligence (AI) and machine learning (Cancho and Sole, 2001; Sigman and Cecchi, 2002).

The results were remarkable. In Judeo-Christian texts, similitude to introspection increased monotonically over time, with a big change in slope from the Old to the New Testaments. In Greco-Roman texts, comprising 53 authors from Homer to Julius Cesar, a more complex dynamics appeared, with increases in similitude to introspection through periods of cultural development, and decreases during periods of cultural decadence. Contemporary texts showed overall increase, with periods of decline prior to and during the two World Wars. As Jaynes would have predicted, the rise and fall of entire societies seems to be paralleled by increases and decreases in introspection, respectively.

Diuk et al. show that the evolution of mental life can be quantified from the cultural record, opening a whole new avenue of hypothesis testing for Jaynes' theory. While it is impossible to prove that pre-Axial people “heard” the voices of the gods, the findings suggest new ways of studying historical and contemporary texts. In particular, the probing of ancient texts with words like “dream,” “god” and “hallucination” has great potential to test Jaynesian concepts.

The featured study lends supports to the notion that consciousness is a social construct in constant flux. Quoting senior author Guillermo Cecchi, “it is not just the “trending topics,” but the entire cognitive make-up that changes over time, indicating that culture co-evolves with available cognitive states, and what is socially considered dysfunction can be tested in a more quantitative way.” Indeed, computer science tools are now being applied to psychology and psychiatry (Gottschalk and Bechtel, 2005; Strous et al., 2009; Cabana et al., 2011; Cohen and Elvevåg, 2014; Valle-Lisboa et al., 2014). Graph-theoretical analysis of speech from psychotic patients leads to accurate differential diagnosis of schizophrenia and bipolar disorder type I (Mota et al., 2012, 2014). Semantic analysis is likely to yield even better classification of psychopathological speech symptoms, as indicated by the successful discrimination of mental states induced by psychoactive drugs (Bedi et al., 2014).

The quantification of behavior and the availability of big data create new opportunities in neuroscience research. For instance, there are now many more tools to study the interplay between language, literacy, introspection and lateralization. The featured study opens especially useful paths for the recent boom in the decoding of neural activity, through which abstract concepts can be directly measured (Kay et al., 2008; Mitchell et al., 2008; Horikawa et al., 2013). Cortical semantic representations were recently found to be warped by attention in a holistic manner (Çukur et al., 2013).

More speculatively, the featured study suggests a more intimate relationship between AI and neuroscience, i.e., AI as a tool used to understand the brain and the mind, and not just to mimic the mind. Given the brain's complexity, this may be the only way forward. Without an objective way to model and quantify meaning, no deep understanding of the mind will be possible. A bold step in this direction is precisely what the discoveries by Diuk, Slezak and collaborators contribute.

## Conflict of interest statement

The author declares that the research was conducted in the absence of any commercial or financial relationships that could be construed as a potential conflict of interest.
